# Honokiol Induces Calpain-Mediated Glucose-Regulated Protein-94 Cleavage and Apoptosis in Human Gastric Cancer Cells and Reduces Tumor Growth

**DOI:** 10.1371/journal.pone.0001096

**Published:** 2007-10-31

**Authors:** Meei Ling Sheu, Shing Hwa Liu, Keng Hsin Lan

**Affiliations:** 1 Institute of Medical Technology, National Chung Hsing University, Taichung, Taiwan; 2 Department of Education and Research, Taichung Veterans General Hospital, Taichung, Taiwan; 3 Institute of Toxicology, College of Medicine, National Taiwan University, Taipei, Taiwan; 4 Department of Surgery, National Taiwan University Hospital, Taipei, Taiwan; 5 Division of Gastroenterology, Department of Medicine, National Yang-Ming University, Taipei, Taiwan; Cairo University, Egypt

## Abstract

**Background:**

Honokiol, a small molecular weight natural product, has been shown to possess potent anti-neoplastic and anti-angiogenic properties. Its molecular mechanisms and the ability of anti-gastric cancer remain unknown. It has been shown that the anti-apoptotic function of the glucose-regulated proteins (GRPs) predicts that their induction in neoplastic cells can lead to cancer progression and drug resistance. We explored the effects of honokiol on the regulation of GRPs and apoptosis in human gastric cancer cells and tumor growth.

**Methodology and Principal Findings:**

Treatment of various human gastric cancer cells with honokiol led to the induction of GRP94 cleavage, but did not affect GRP78. Silencing of GRP94 by small interfering RNA (siRNA) could induce cell apoptosis. Treatment of cells with honokiol or chemotherapeutics agent etoposide enhanced the increase in apoptosis and GRP94 degradation. The calpain activity and calpain-II (m-calpain) protein (but not calpain-I (µ-calpain)) level could also be increased by honokiol. Honokiol-induced GRP94 down-regulation and apoptosis in gastric cancer cells could be reversed by siRNA targeting calpain-II and calpain inhibitors. Furthermore, the results of immunofluorescence staining and immunoprecipitation revealed a specific interaction of GRP94 with calpain-II in cells following honokiol treatment. We next observed that tumor GRP94 over-expression and tumor growth in BALB/c nude mice, which were inoculated with human gastric cancer cells MKN45, are markedly decreased by honokiol treatment.

**Conclusions and Significance:**

These results provide the first evidence that honokiol-induced calpain-II-mediated GRP94 cleavage causes human gastric cancer cell apoptosis. We further suggest that honokiol may be a possible therapeutic agent to improve clinical outcome of gastric cancer.

## Introduction

Gastric cancer is the second most common cause of cancer death in the world [1]. Almost two-thirds of the cases occur in developing countries and 42% in China alone [1], [2]. The molecular targets and mechanisms underlying poor prognosis are not well understood. The treatment of locally advanced gastric cancer remains a major challenge. Despite recent advances in treatment, the clinical outcome for gastric cancer patients remains poor. Apart from surgery, the role of adjuvant therapy remains unproven [2], [3]. Thus the need to identify potential novel therapeutic and chemopreventive agents is obvious.

Honokiol, an active component isolated and purified from the *Magnolia officinalis*, has been demonstrated to possess the effects of anti-oxidation [4], against lipid peroxidation [5] and anti-inflammatory *in vitro* and *in vivo* [6], [7]. Honokiol has also been shown to be a systemically available and non-toxic inhibitor of angiogenesis [8]. The recent studies reported that honokiol induces caspase-dependent apoptosis in B-cell chronic lymphocytic leukemia cells and overcomes conventional drug resistance in human multiple myeloma by induction of caspase-dependent and -independent apoptosis [9], [10]. Although a full assessment of the clinical potential of the compounds is not yet possible, the pharmacokinetics of honokiol has been thoroughly investigated, revealing that honokiol is available upon clinical cancer therapy. More natural products containing a variety of therapeutic compounds useful in preventing the development of malignancies continue to be discovered; however, very little is known about their underlying mechanisms of action or their molecular target.

Glucose-regulated protein (GRP)94 is a most abundant glycoprotein in endoplasmic reticulum (ER) and only be identified in vertebrate. Over-expression antisense and ribozyme approaches in tissues culture systems directly demonstrated that GRP78 and GRP94 could protect cells against death [11]–[13]. The protective function of the GRPs has also been observed in resistance to radiation in cervical cancer [14]. The anti-apoptotic function of the GRPs also predicts that their induction in neoplastic cells can lead to cancer progression and drug resistance [15]–[18]. Pathological conditions such as tumor growth and malignancy have been suggested to correlate with cytoprotective protein GRP94 over-expression [19]. In metastatic malignancies model, a significant efficacy of the GRP94-based gene/immunotherapy strategy was shown when it was combined with radiation therapy [20]. The ER plays a direct role in activating a subset of caspase during activation of apoptosis that occurs during ER stress [21]. On the other hand, calpains are a family of Ca^2+^-dependent intracellular cysteine proteases. Ubiquitously expressed calpain-I (μ-calpain) and calpain-II (m-calpain) proteases are implicated in development of apoptosis. A recent study has shown that ubiquitous calpains promote caspase-12 and JNK activation during ER stress-induced apoptosis [22]. It has also been indicated that GRP94 with Ca^2+^-binding and anti-apoptotic properties is a proteolytic target of calpain during etoposide-induced apoptosis [23]. Moreover, in several experimental models of apoptosis, it has been shown that the amino-terminal calpain inhibitory unit of calpastatin can be cleaved by caspases, suggesting the cleavage is essential for the regulation of calpain activity during cell death [24]–[28]. Caspase-7, which is recruited to the ER in stressed cells, may likewise cleave and activate caspase-12 [29]–[31]. The effects of honokiol on the GRPs-related signaling and apoptosis still remain unknown. In the present study, we explored the molecular mechanisms of honokiol on human gastric cancer cell apoptosis and tumor growth *in vitro* and *in vivo*. Unexpectedly, we found that GRP94 undergoes specific proteolytic cleavage by calpain during honokiol-induced apoptosis. These observations may give evidences that honokiol is a possible therapeutic agent to improve clinical outcome of gastric cancer.

## Results

### Kinetics of GRP94 proteolytic cleavage in human gastric cancer cell lines treated with honokiol

**Figure 1:**
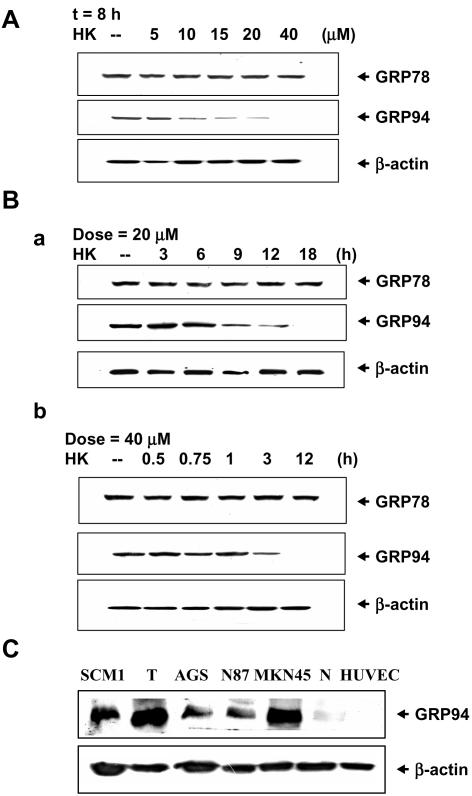
Effects of honokiol on the expressions of GRP94 and GRP78 in human gastric cancer cell lines. (A) Western blot analyses for GRP94 and GRP78 in MKN45 cells treated with honokiol for 8 h in a dose-response manner. (B) Western blot analyses for GRP94 and GRP78 in MKN45 cells treated with honokiol (a, 20µM; b, 40 µM) in a time-response manner. (C) Comparison of GRP94 protein expression among human gastric cancer cell lines (SCM-1, AGS, N87, and MKN45), tumor isolated from MKN45 cells-inoculated mice (T), normal mouse gastric epithelium tissue (N), and human umbilical vein endothelial cells (HUVEC). All results shown are representative of at least four independent experiments.

**Figure 2:**
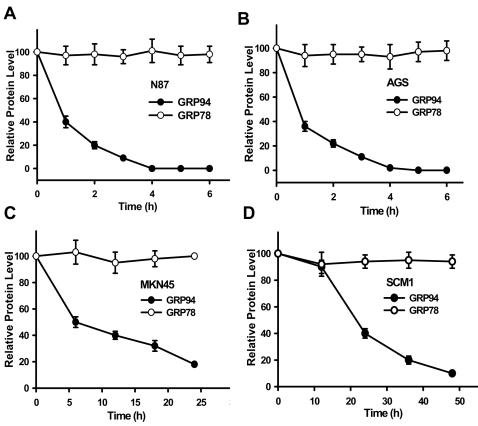
Kinetics changes of GRP94 and GRP78 protein expression in various human gastric cancer cell lines following honokiol treatments. Western blot analyses for GRP94 and GRP78 in cells (N87 (A), AGS (B), MKN45 (C) and SCM-1 (D)) treated with 40 µM honokiol for various time courses as indicated. Data are presented as mean±SEM (n = 5).

We firstly examined the effects of honokiol on the expressions of GRP94 and GRP78 in various human gastric cancer cell lines. Honokiol markedly decreases the levels of GRP94 in a dose- and time-dependent manner, but GRP78 levels are not affected (Figs. 1 and 2). Kinetics of honokiol-induced GRP94 cleavage in various human gastric cancer cells were shown in Fig. 2. MKN45 or SCM-1 cells were more resistant to honokiol-induced responses than AGS or N87 cells; the levels of GRP94 were reduced to about 50% for twice as much time. The levels of GRP78 proteins remain unchanged in all four gastric cancer cell lines. Moreover, there are little GRP94 protein expressions in normal mouse gastric epithelium tissue and human endothelial cells as compared with gastric cancer cells (Fig. 1C).

### Honokiol induces GRP94 cleavage–associated apoptotic response

**Figure 3:**
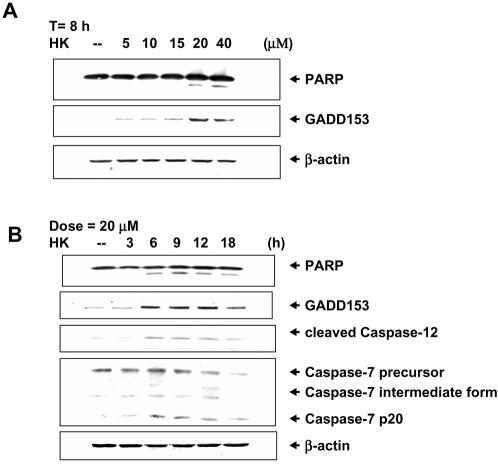
Honokiol induces a GRP94 degradation-associated apoptotic response in human gastric cancer cells. (A) Western blot analyses for PARP and GADD153 in MKN45 cells treated with honokiol for 8 h in a dose-response manner. (B) Time course responses for PARP, GADD153, caspase-7 and caspase-12 in MKN45 cells treated with honokiol (20 µM). Results shown are representative of at least four independent experiments.

**Figure 4:**
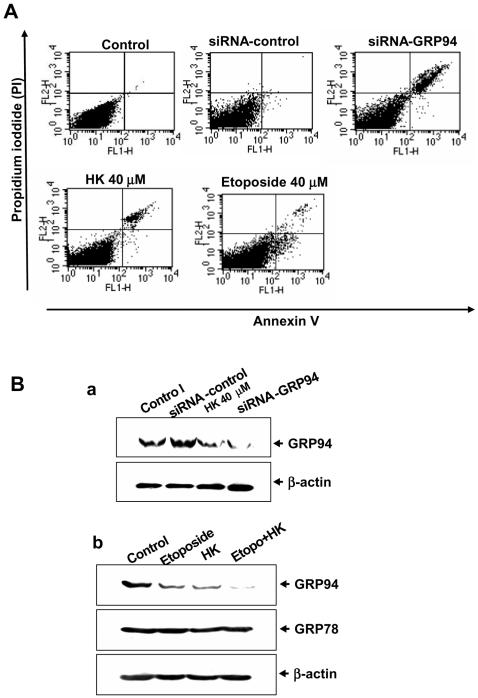
Silencing of GRP94 by siRNA induces cell apoptosis. (A) Apoptosis in GRP94-siRNA-transfected MKN45 cells was analyzed by Annexin V/PI staining as described under Materials and Methods, which was compared with cells treated with honokiol (40 µM) or etoposide (40 µM). (B-a) GRP94 protein levels were detected by Western blot analysis in control and GRP94-siRNA-transfected MKN45 cells. (B-b) Honokiol and etoposide induce GRP94 cleavage in MKN45 cells. Cells were treated with 20 µM honokiol and 40 µM etoposide (Etopo.) as indicated for 4 h. Results shown are representative of at least four independent experiments.

As shown in Fig. 3, honokiol increased the poly(ADP-ribose) polymerase (PARP) cleavage and DNA damage inducible gene CHOP/GADD153 (a protein has been identified to mediate ER stress-induced apoptosis) levels in MKN45 cells in a dose- and time-dependent manner. Honokiol 20 and 40 M also triggered the expressions of cleaved caspase-12 and caspase-7 (p20), which the magnitude of increase was proportional to the timing (Fig. 3B). Moreover, to understand whether GRP94 cleavage was involved in the human gastric cancer cell apoptosis, apoptosis was detected in GRP94-siRNA-transfected cells. Silencing of GRP94 by siRNA induced cell apoptosis (Fig. 4A) and decreased GRP94 protein expression (Fig. 4B-a). We also compared the effects of honokiol on apoptosis and GRP94 degradation in human gastric cancer cells with chemotherapeutics agent etoposide. The results showed that treatment of cells with honokiol or etoposide enhanced the increase in apoptosis (Fig. 4A) and GRP94 degradation (Fig. 4B-b). When cells were simultaneously treated with 40 M etoposide and 20 M honokiol, a larger increase in GRP94 degradation than they did was shown (Fig. 4B-b); these results imply that the combining of honokiol with other anticancer drugs may be a potential therapeutic strategy.

### Calpain is required for honokiol-mediated cell apoptosis

**Figure 5:**
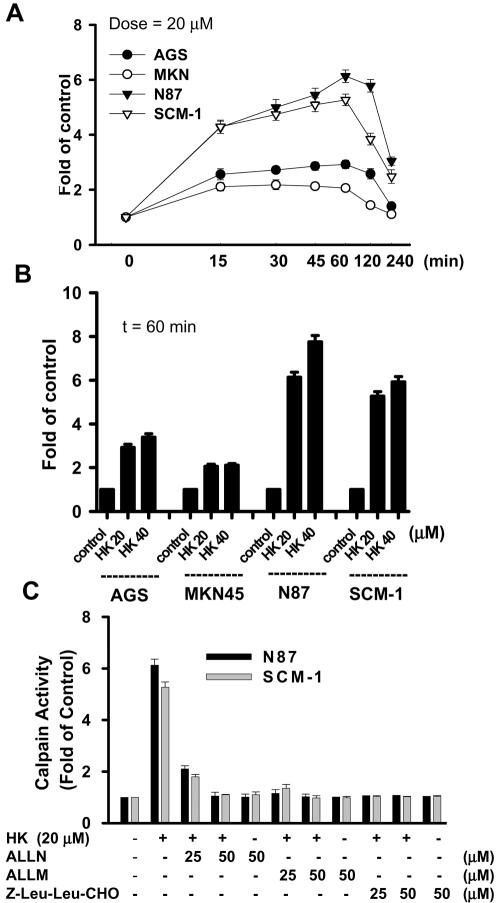
Honokiol activates calpain activity. Calpain activity was measured with the fluorescent calpain substrate Suc-LLVY-AMC in N87, AGS, MKN45 and SCM-1 cells. (A) Time course responses to honokiol (20 µM) treatment. Data are expressed in terms of fold of control conditions. (B) Honokiol (20 and 40 µM) increases calpain activity at 60 min treatment. (C) Calpain inhibitors ALLN, ALLM, and Z-Leu-Leu-CHO; 25 and 50 µM) significantly inhibited honokiol-increased calpain activity. Data are presented as mean±SEM (n = 4).

We next determined whether the activity of calpain (calcium-dependent thiol proteases) would be induced by honokiol in four gastric cancer cell lines. As shown in Fig. 5A, honokiol 20 M increased calpain activity in a time-dependent manner, which started to increase at 15 min, and peaked at 60 min, and then dropped down to a lower level at 4 h. Cells remained adherent over the time course, with no loss of viability (data not shown). In Fig. 5B, the results showed that the increase of calpain activity in response to honokiol (20 and 40 M) for 60 min in four gastric cancer cell lines. Furthermore, calpain inhibitors, N-acetyl-leu-leu-norleucinal (ALLN), N-acetyl-leu-leu-methioninal (ALLM), and Z-Leu-Leu-CHO, effectively inhibited the increase of calpain activity induced by honokiol in N87 and SCM-1 cells (Fig. 5C).

**Figure 6:**
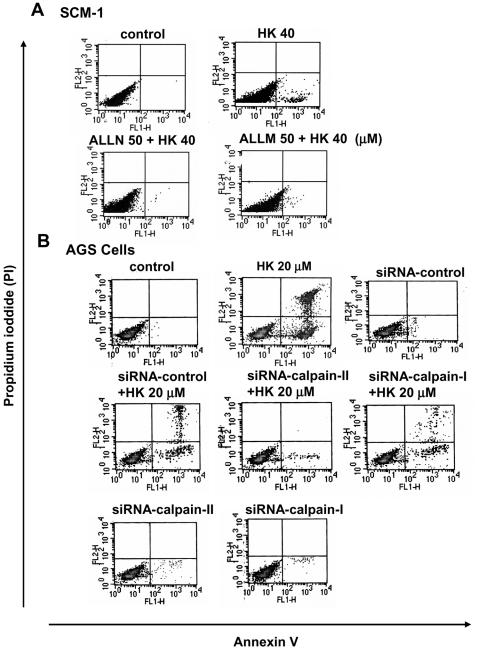
Suppression of calpain activity by calpain inhibitors or c siRNA-calpain-II decreased honokiol-induced cell apoptosis. Human gastric cancer cells were analyzed for apoptosis by Annexin V/PI staining as described under Materials and Methods. (A) SCM-1 cells were treated with honokiol (HK, 40 µM) for 4 h in the presence or absence of calpain inhibitors (ALLN and ALLM, 50 µM). (B) AGS cells transfected with siRNA-calpain-I- or siRNA-calpain-II were treated with honokiol (HK, 20 µM) for 4 h. Results shown are representative of at least four independent experiments.

**Figure 7:**
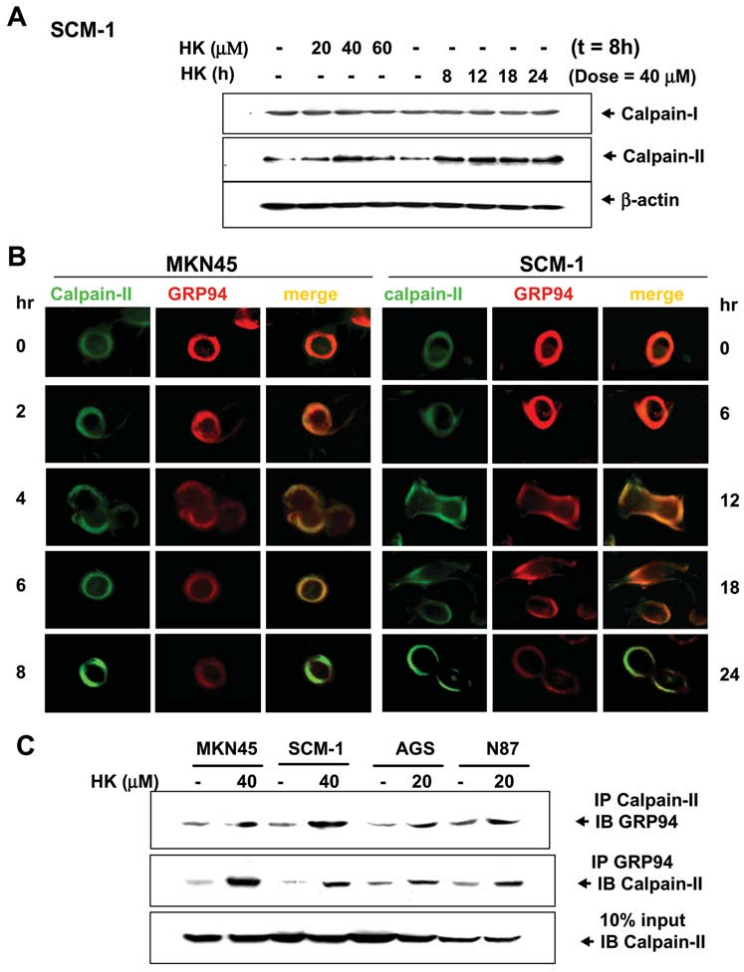
Effects of honokiol on calpain-I and II protein levels and interaction of calpain and GRP94. Cells were treated with honokiol (20–60 µM) for various time courses as indicated. (A) Calpain-I and II protein levels were detected by Western blot analysis in honokiol-treated SCM-1 cells. (B) Primary antibodies for calpain-II and GRP94 were applied to the cells (MKN45 and SCM-1) followed by secondary antibodies coupled with FITC-conjugated or TRITC-conjugated, respectively. Co-localization of two labeled antigens was detected as a single image when the images from both channels were overlaid. (C) Interaction of calpain and GRP94 were detected in N87, AGS, MKN45 and SCM-1 cells. Immunoprecipitated proteins were collected and subjected to SDS-PAGE and immunoblotting with anti-calpain-II or anti-GRP94 antibodies. Results shown are representative of at least four independent experiments.

We further tested whether decreased calpain activity could compromise the ability of honokiol on apoptosis induction. As shown in Fig. 6A, honokiol (40 M) induced cell apoptosis in SCM-1 cell, which could be reversed by calpain inhibitors (ALLN or ALLM). Moreover, honokiol enhanced calpain-II protein but not calpain-I protein expressions in SCM-1 cells (Fig. 7A). Using a siRNA approach to knockdown calpain-II activity led to a significant abatement of honokiol (20 M)-induced apoptosis in human gastric cancer cells after 4 h treatment (Fig. 6B). SiRNA-calpain-I did not affect honokiol-induced apoptosis (Fig. 6B).

### Localization and interaction of calpain-II and GRP94 following honokiol treatment

**Figure 8:**
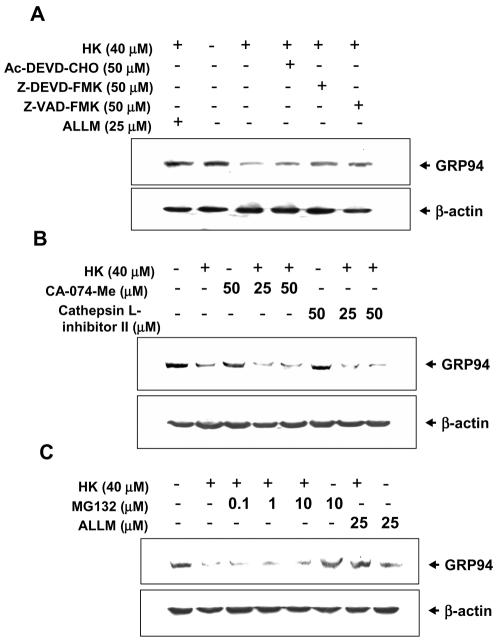
Effect of inhibitors of caspases, proteasome and cathepsins on honokiol-induced GRP94 cleavage in SCM-1 cells. Cells were either untreated or pretreated with inhibitors for 1 h followed by 40 µM honokiol treatment for another 24 h. (A) caspase inhibitor (Z-VAD-FMK, 25 and 50 µM) or calpain inhibitor (ALLN, 25 µM); (B) cathepsin B inhibitor (CA-074-Me, 25 and 50 µM) or cathepsin L inhibitor (cathepsin L inhibitor II, 25 and 50 µM); (C) proteasome inhibitor (MG132, 0.1–10 µM) or calpain inhibitor (ALLN, 25 µM). Results shown are representative of at least four independent experiments.

The next step was to elucidate the role of calpain-II in honokiol-induced GRP94 degradation. In laser confocal microscopic study, the localization of calpain-II protein was determined by green fluorescence, whereas the localization of GRP94 was determined by red fluorescence. As shown in Fig. 7B, both calpain-II and GRP94 were detected in cytoplasm with co-localization in human gastric cancer cells. The fluorescence of calpain-II was gradually increased, but fluorescence of GRP94 was gradually decreased in human gastric cancer cells under honokiol treatment. To further confirm the results from immunocytochemical staining that calpain-II interacts with GRP94, the tests of co-immunoprecipitation and Western blotting in gastric cancer cells were performed. As shown in Fig. 7C, calpain-II was specifically associated with GRP94 in various gastric cancer cells in the presence of honokiol (20 and 40 M) as compared with IgG control. Moreover, we tested whether the proteolytic activities of caspase, proteasome or cathepsin were involved in the cleavage of GRP94. Our results showed that treatment of cells with caspase inhibitors (Ac-DEVD-CHO, Z-VAD-FMK and Z-DEVD-FMK, 50 M) at high dose partially blocked the cleavage of GRP94 during honokiol-induced apoptosis (Fig. 8A) as compared with calpain inhibitor ALLM 25 M, which could completely block GRP94 cleavage. Pretreatment of cells with the cathepsin B inhibitor CA-074-Me 25 and 50 M and cathepsin L inhibitor cathepsin L inhibitor II 25 and 50 M and proteasome inhibitor MG132 0.1-10 M was unable to block GRP94 cleavage (Figs. 8B and 8C). Furthermore, the specific cleavage of GRP94 by calpain could be observed using cell lysates prepared from human gastric cancer cells (data not shown).

**Figure 9:**
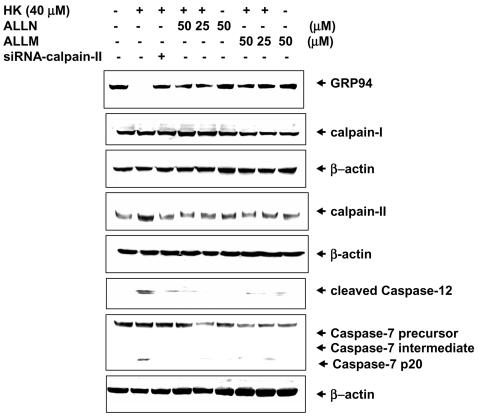
Honokiol induces calpain-mediated GRP94 degradation and apoptosis. Western blotting for determination of GRP94 degradation and calpain-I and II expression and activation of caspase-7 and caspase-12 in SCM-1 cells 24 h after honokiol (40 µM) treatment in the presence or absence of calpain inhibitors (ALLN and ALLM, 25 and 50 µM) was detected. In some experiments, SCM-1 cells were transfected with calpain-II-siRNA or control-siRNA. Results shown are representative of at least four independent experiments.

We next investigated whether the calpain activation is required for honokiol-induced cell apoptosis. In Fig. 9, the increases of calpain-II protein expression and GRP94 degradation and caspase-7 and caspase-12 activation induced by honokiol (40 M) were prevented by pharmacological calpain inhibitors and transient transfection of siRNA-calpain-II in SCM-1 cells.

### Honokiol attenuates tumor GRP94 over-expression and tumor growth in nude mice

**Figure 10:**
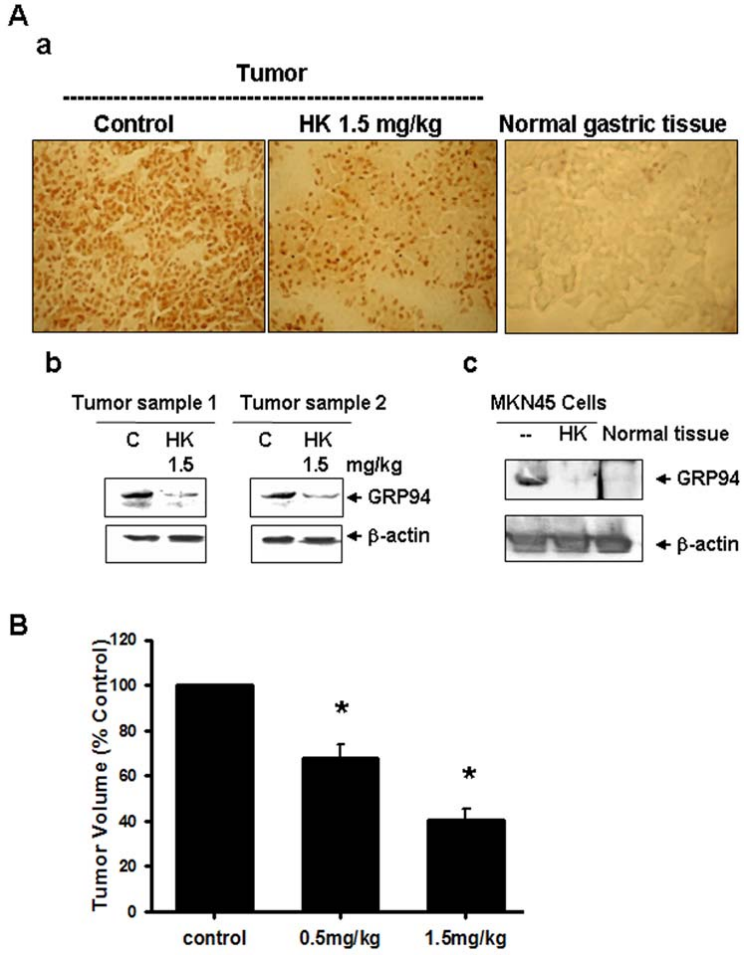
Honokiol reduces tumor GRP94 over-expression and tumor growth. Tumors in nude mice were established for 14 days after MKN45 cells injection and followed by treatment with 0.5 and 1.5 mg/kg honokiol every day for 10 days. (A-a) GRP94 expressions in tumors or normal gastric tissues were determined by immunohistochemistry. The sections were stained with GRP94 antibody as described under Materials and Methods. The expressions of GRP94 proteins in cytoplasm were stained into dark brown. (A-b) Western blotting for determination of GRP94 proteins in tumors with or without honokiol treatment was detected. (A-c) The detection of GRP94 protein expression by Western blotting in MKN45 cells with or without honokiol (40 µM) treatment or in normal gastric tissues was shown. Results shown are representative of at least four independent experiments. (B) Tumor volume was assessed. Data are presented as mean±SEM (n = 7).

Nude mice were inoculated with 4×10^6^ undifferentiated adenocarcinoma cells MKN45 and treated with honokiol 0.5 and 1.5 mg/kg or vehicle when tumor became evident. Immunohistological and Western blotting analysis showed that GRP94 over-expression and accumulated in tumor region, but not in normal gastric tissue (Figs. 10A-a and 10A-b). Honokiol markedly decreased the accumulation of GRP94 in tumors as compared with vehicle control (Figs. 10A-a and 10A-b). The expression of GRP78 was not affected (data not shown). To determine whether honokiol could suppress tumor growth *in vivo*, solid tumors were established in mice and the anti-tumor effect of repeatedly injected honokiol was studied. As shown in Fig. 10B, the administration of honokiol 0.5 and 1.5 mg/kg showed a significant anti-tumor activity.

## Discussion

We reported here for the first time that calpain, a nonlysosomal Ca^2+^-activated cysteine protease, especially calpain-II, plays a key role in the cleavage of GRP94, but GRP78 is not affected, and in the regulation of apoptosis induced by honokiol in human gastric cancer cells. In particular, we provided functional evidences that the cleavage of GRP94 upon honokiol treatment acts as a therapeutic benefit, inhibiting tumor growth in mouse model of human gastric carcinoma. To our knowledge, this is the first report to show that honokiol can manipulate calpain-inducible chaperone protein GRP94 degradation being the target during apoptosis.

Studies from multiple laboratories pointed to the ER as a target compartment by therapeutic agents implicated in apoptosis execution. Cytosolic Ca^2+^ has been implicated as a second messenger of proapoptosis involved in both triggering apoptosis and regulating caspases or calpains [32]–[34]. A recent study has shown that calpain is an important mediator of resveratrol (a natural plant polyphenol)-induced apoptosis in breast cancer [35]. They found that resveratrol can cause an increase in intracellular Ca^2+^, and activates calpain-mediated apoptosis, leading to the degradation of plasma membrane Ca^2+^-ATPase isoform 1 and fodrin in caspase-3 deficient MCF-7 cells. Honokiol has also been reported to induce Ca^2+^ mobilization in rat cortical neurons and human neuroblastoma SH-SY5Y cells, presumably through the activation of phospholipase C and IP_3_ receptors [36]. In the present study, we found that honokiol is capable of increasing calpain activity and calpain-II protein expression but not calpain-I during honokiol-induced gastric cancer cell apoptosis. Moreover, calpain inhibitors and silencing of calpain-II by siRNA significantly reversed the honokiol-induced apoptosis. These findings imply that Ca^2+^-triggered calpain-II activation may play a potential role in honokiol-induced apoptosis.

GRP94/gp96 is the ER resident member of the HSP90 family. GRP94 plays an important role in maintaining cellular homeostasis. This conventional concept of GRP94 as protein folding chaperone is updated by the discoveries that GRPs promote tumor proliferation, metastasis, drug resistance, and immunotherapy, which have major clinical implications in the prognosis and treatment of cancer [3]. GRP94 has been demonstrated to be associated with tumorigenicity in cancer cell lines, rodent tumor models and human cancer biopsies [37]–[39]. This is consistent with the findings that the induction of GRPs causes a protective function as the survival responses to nutrient starvation, acidosis, and hypoxia conditions, which are common in poorly vascularized solid tumors [11], [40]. It has also been appeared that most of the GRPs in tumor cells is engaged in multi-chaperone complexes, while it is not in normal cells [41]. Vaccination of mice with tumor-derived stress proteins, GRP94, can elicit anti-tumor immune responses, yielding a marked suppression of tumor growth and metastasis. The activity of Hsp90 inhibitors has been well validated in preclinical breast cancer models, both in single-agent studies and in combination with conventional chemotherapy [41]. Moreover, it has been suggested that GRP94 is a physiological substrate for calpain [23]. Calpain has been demonstrated to be activated at the ER membrane, where it interacts with GRP94, resulting in its specific proteolytic cleavage as the cells undergo apoptosis triggered by etoposide [23]. In current study, we found that honokiol-induced GRP94 cleavage and apoptosis in human gastric cancer cells can be completely reversed by calpain inhibitors and silencing of calpain-II by siRNA. Caspase inhibitors at high dose partially reversed the honokiol-induced GRP94 cleavage. Pretreatment of cells with the cathepsin B and L inhibitors and proteasome inhibitor was unable to block honokiol-induced GRP94 cleavage. We also demonstrated that silencing of GRP94 by siRNA can induce gastric cancer cell apoptosis. Furthermore, the results of immunocytochemical staining and immunoprecipitation revealed a specific interaction of GRP94 with calpain-II in gastric cancer cells following honokiol treatment. The specific cleavage of GRP94 by calpain could also be observed using cell lysates prepared from human gastric cancer cells. These findings, therefore, suggest that calpain-II-mediated GRP94 cleavage plays an important role in honokiol-triggered gastric cancer cell apoptosis.

Calpains and caspases are two families of cysteine proteases that they are involved in regulating pathological cell death [22], [31]. These proteases share several death-related substrates including caspases themselves, cytoskeletal proteins, Bax, and Bid [42]. Calpain-mediated proteolysis proceeds in a limited manner but does not require a specific amino acid residue like that of caspases. Although both calpain and caspase have been proposed to play important roles in regulating pathological cell death, the interactions of these two families of proteases under pathological conditions are not clear. In the present study, knockdown and pharmacological inhibitors approaches have contributed significantly to our knowledge of calpain biology, particularly with respect to its specific function on cell apoptosis, which is possible that caspases 7 and 12 are downstream from calpain in mediating honokiol-induced gastric cancer cell apoptosis.

Natural product drugs have been suggested to play a dominant role in pharmaceutical care [43]. Natural products are one of the important sources of potential cancer chemotherapeutic and chemopreventive agents [43]. Honokiol has been widely used in the traditional Chinese and Japanese medicine for several thousand years, mainly, for the treatment of anti-thrombocytic, anti-bacterial, anti-inflammatory, and anxiolytic effects. Previous reports have demonstrated that honokiol is also possessing potent anti-neoplastic and anti-angiogenic properties [6], [19], [38]. However, the precise molecular mechanism of exhibited anti-tumor activity by honokiol is not well understood. Thus, the results of this study provide evidences for the anti-tumor activity of honokiol in gastric cancer, and more importantly, the molecular basis for its effect. We found that honokiol induces the activation of calpain, GRP94 cleavage, and apoptosis in human gastric cancer cells. Moreover, after administration of honokiol in nude nice implanted with MKN45 gastric tumor cells showed significant anti-tumor activity. This preclinical study can serve as a framework and represents a promising novel targeted approach. In addition, the natural compounds have been shown to combine with conventional cytotoxic agents may be clinical beneficial with anticancer drugs. This advantage plus the emerging evidence of its anti-tumor mechanisms make honokiol ongoing clinical application for being an effective and safe anti-tumor agent.

## Materials and Methods

### Cells, Culture Conditions and Reagents

Human cell lines including Caucasian gastric cancer cell lines (AGS, a moderately-poorly differentiated adenocarcinoma cell line, and N87, a well differentiated carcinoma cell line) and Asian gastric cancer cell lines (MKN45 and SCM-1, the undifferentiated adenocarcinoma cells) were obtained from cell bank in cancer center of Taipei veterans general hospital (Taiwan). Cells were maintained in RPMI 1640 medium containing 10% heat-inactivated FCS (Life Technologies) and streptomycin/penicillin (Life Technologies) in a humidified 5% CO_2_ atmosphere. Honokiol was obtained from Wako Chemical Company (Japan), and its purity was determined to be a minimum of 99% by high-performance liquid chromatography.

### Western Blot Analysis and Immunoprecipitations

Whole cell lysates were prepared as described previously [44]. Proteins were separated by pre-cast 8–20% SDS-polyacrylamide gel electrophoresis, and then electrophoretically transferred from the gel onto polyvinylidene difluoride membranes. After blocking, blots were incubated with, anti-GRP94, anti-GRP78, anti-caspase 12, anti-caspase 7, anti-PARP, anti-GADD153 (Santa Cruz Biotechnology), anti-calpain-I (µ-calpain), anti-calpain-II (m-calpain) (Santa Cruz Biotechnology), and anti-β actin (Sigma) antibodies in PBS within 0.1% Tween 20 for 1 h followed by three 10 min washes in PBS within 0.1% Tween 20. The membranes were then incubated with horseradish peroxidase-conjugated secondary antibodies for 60 min. In immunoprecipitation, proteins (500 g) were incubated with specific antibodies and immobilized onto protein A-Sepharose beads. Beads were washed extensively with Bacco′s immunoprecipitative buffer, boiled, and microcentrifuged. Input 10% of cell lysate for IP and 50 g proteins were analyzed by Western blotting. Detection was performed with Western blotting reagent ECL (Amersham), and chemiluminescence was exposed by the Kodak X-Omat films.

### Calpain Activity Assays

Suc-Leu-Leu-Val-Tyr-AMC is a calpain protease substrate. Quantitation of 7-amino-4-methylcoumarin (AMC) fluorescence permits the monitoring of enzyme hydrolysis of the peptide-AMC conjugate and can be used to measure enzyme activity. Cells were prepared and treated on 24-well Corning/Costar plates. Prior to addition of inhibitors cells were loaded with 40 M Suc-Leu-Leu-Val-Tyr-AMC (Biomol) and treated with honokiol for indicated timing at 37°C in a humidified 5% CO_2_ incubator. Proteolysis of the fluorescent probe was monitored using a fluorescent plate reading system (HTS-7000 Plus Series BioAssay, Perkin Elmer) with filter settings of 360±20 nm for excitation and 460±20 nm for emission.

### SiRNA and Transfection Assays

The siRNA duplexes specific for the inhibition of calpain-I (µ-calpain) and calpain-II (m-calpain) expressions in human cells were obtained from Santa Cruz Biotechnology: calpain-I, catalog no. sc-29885, a pool of 3 target-specific 20–25 nt siRNAs; calpain-II, catalog no. sc-41459, a target-specific 20–25 nt siRNA. The control siRNA (catalog no. sc-37007) is a non-targeting 20–25 nt siRNA designed as a negative control. Moreover, the siRNA duplexes specific for the inhibition of GRP94 expression was synthesized commercially by MWG Biotech (Germany). The sense (top) and antisense (bottom) sequences of the GRP94 siRNA duplex were as follows: 5′-GAAGAAGCAUCUGAUUACCTT-3′; 3′-TTCUUCUUCGUAGACUAAUGG-5′. As a nonspecific control, a NC siRNA duplex with random sequences was designed as follows: 5′-AGUUCAACGAGUAUCAGCATT-3′; 3′-TTUCAAGUUGCUCAUAGUCGU-5′. The siRNAs were used at a concentration of 100–200 nM for transient transfection of cells with Lipofectin (Invitrogen) per well in a 6-well plate with fresh medium. After 24 h from the initial transfection and 24–36 h treatment, cells or cell lysates were collected and analyzed for apoptosis or protein expression, respectively.

### Immunofluorescence and Laser Scanning Confocal Microscopy

Cells were washed twice with PBS, fixed in 4% paraformaldehyde for 30 min and then blocked by incubation in 1% bovine serum albumin in PBS. Primary antibodies as indicated were applied to the slides at a dilution of 1∶500 and incubated at 4°C overnight. The samples were treated with FITC-conjugated or TRITC-conjugated secondary antibodies (Sigma). The FITC-labeled or TRITC-labeled cells were then analyzed by fluorescence microscopy. For laser scan microscopy, immunofluorescence-labeled cells were analyzed using an inverted laser scanning microscope (Zeiss LSM 410 invert, Germany), equipped with both argon ion (488 nm) and HeNe (543 nm) lasers. Co-localization of two labeled antigens was detected as a single image when the images from both channels were overlaid. Confocal images were background-subtracted and merged using the Confocal Assistant software program, and processed with Adobe Photoshop software.

### Annexin-V FITC and PI Double Staining

The annexin V/propidium iodide (BD Clontech) was used to quantify numbers of apoptotic cells as described previously [44]. Cells were washed twice with PBS and stained with annexin V and PI for 20 min at room temperature. The level of apoptosis was determined by measuring the fluorescence of the cells by flow cytometer (Becton Dickinson). Data acquisition and analysis were performed by the CellQuest program (Becton Dickinson).

### Animals

Animal experiments were carried out according to the guidelines issued by the Committee of Animal Experiments, National Chung Hsing University, Taichung, Taiwan. Male BALB/c nude mice (*nu/nu*) were purchased from NLAC Taiwan, Inc., Taipei, Taiwan. The mice were bred and maintained under specific pathogen–free conditions, provided with sterilized food and water ad libitum, and housed in a barrier facility with 12 h light/dark cycles. Experimental procedures were done on 6-week-old mice. The animals were euthanized when they appeared moribund.

### Tumor Growth Assay

Cell tumorigenicity was examined in 6-week-old male BALB/c nude mice. Briefly, 4×10^6^ of cells (MKN45) in an exponential growth phase were suspended in 1 ml culture medium with 10% fetal bovine serum. A single-cell suspension of MKN45 was subcutaneously inoculated into the dorsal side of the mice. Tumors were established for 14 days after MKN45 cells inoculation and followed by treatment with vehicle, 0.5 and 1.5 mg/kg honokiol every day for 10 days (7 mice/group). The volume of the implanted tumor in dorsal side of mice was measured twice a week with a caliper, using the formula *V* = (*LW* ^2^) π/6: where *V*, volume (mm^3^); *L*, biggest diameter (mm); *W*, smallest diameter (mm).

### Immunohistochemistry

The expression of GRP94 in mouse solid tumor was examined by immunohistochemistry. Thin tumor sections (5 µm) were prepared, fixed with cold acetone, blocked with 3% hydrogen peroxidase, and stained with for primary GRP94 antibody.

### Statistical Analyses

The values given in this study are presented as mean±SEM. All analyses were performed by analysis of variance followed by a Fisher's least significant difference test. *P* value of less than 0.05 was viewed as statistical significance.
